# Effects of active action observation on cognitive, emotional, motor, and somatosensory outcomes in adolescents with juvenile idiopathic arthritis: a prospective exploratory case series

**DOI:** 10.3389/fnhum.2026.1766070

**Published:** 2026-02-27

**Authors:** Enrique Carrasco-González, Guillermo Ceniza-Bordallo, Daniel Clemente Garulo, Clara Udaondo Gascón, Sergio Lerma-Lara, Roy La Touche

**Affiliations:** 1 Physical Therapy Department, CSEU La Salle, Universidad Autónoma de Madrid, Madrid, Spain; 2 Motion in Brains Research Group, CSEU La Salle, Universidad Autónoma de Madrid, Madrid, Spain; 3 Grupo de Investigación en Neuronciencias Aplicadas a la Rehabilitación (GINARE), Alcorcón, Spain; 4 Department of Psychiatry, Center for Health Outcomes and Interdisciplinary Research, Massachusetts General Hospital, One Bowdoin Square, Boston, MA, United States; 5 Department of Psychiatry, Harvard Medical School, Boston, MA, United States; 6 Unidad de Reumatología Pediátrica, Hospital Universitario Niño Jesús, Madrid, Spain; 7 Unidad de Reumatología Pediátrica, Hospital Universitario La Paz, Madrid, Spain; 8 Ciberinfec, Centro de Investigación Biomédica en Red de Enfermedades Infecciosas, Instituto de Salud Carlos III, Madrid, Spain

**Keywords:** exercise, juvenile idiopathic arthiritis, pain, representation techniques, telerehbilitation

## Abstract

**Background:**

Juvenile Idiopathic Arthritis (JIA) is a heterogeneous pediatric rheumatic disease characterized by persistent pain, functional limitations, and psychosocial difficulties that frequently persist despite optimized pharmacological treatment. Active Action Observation (AAO), which involves observing and then executing goal-directed movements, has been shown to engage mirror-neuron and visuomotor networks. This approach has shown promise in neurological and orthopedic rehabilitation, but has not been studied in adolescents with JIA.

**Objective:**

To assess the feasibility, acceptability, and preliminary clinical effects of an eight-week home-based AAO telerehabilitation program in adolescents with JIA.

**Methods:**

A prospective exploratory case series included 10 adolescents with JIA (11–17 years). The participants completed an eight-week AAO protocol delivered via weekly pre-recorded YouTube videos and brief online follow-up sessions. Outcomes were assessed at baseline, week 4, and week 8 and included pain interference (PROMIS), stress (SSI-SM), self-efficacy (GSES), fear of pain (FOPQ-III), Timed Up and Go (TUG), hand-grip strength, 6-min walk test (6MWT), and cervical pressure pain thresholds (PPT). The analyses focused on descriptive statistics, change scores, percentage change, and robust estimators (Hodges–Lehmann, Kendall’s W).

**Results:**

AAO was feasible and well accepted, with no participants withdrawing, and only mild and transient adverse effects reported. From baseline to week 8, the median changes were −42.1% for pain interference, −22.2% for stress, +18.8% for self-efficacy, and −25.7% for fear of pain/movement. While functional mobility (TUG) showed improvement, handgrip strength and 6-min walk test responses were found to be heterogeneous, and cervical PPT exhibited a pattern of increased sensitivity. In line with AAO’s neurocognitive framework, the observed reduction in fear of pain, coupled with increased self-efficacy and reduced stress, may indicate an update in threat-related motor representations. This update disrupts the previously formed “movement = danger” association, thereby enabling safer functional engagement and less pain interference.

**Conclusion:**

AAO delivered at home via telerehabilitation appears to be a feasible, acceptable, and potentially beneficial adjunct for adolescents with JIA, particularly for pain-related and psychological outcomes. These preliminary findings support the feasibility of AAO and justify future controlled trials to establish its efficacy and elucidate the underlying mechanisms.

## 1 Introduction

Juvenile Idiopathic Arthritis (JIA) is a term used to describe all forms of arthritis with onset before the age of 16, persist for at least six weeks, and do not have a known cause (Petty et al., 2015; Ravelli and Martini, 2007; Szer et al., 2006). JIA is diagnosed based on a combination of clinical and laboratory characteristics that represent several distinct but interrelated entities. Some subtypes present with well-defined systemic features, such as fever, rashes, or serositis, while others show more heterogeneous manifestations involving generalized inflammation of both small and large joints (Ravelli and Martini, 2007; Thompson et al., 2010).

Pain is among the most prevalent and disabling symptoms in persons with JIA (PwJIA). Approximately 27% of individuals with JAI report moderate-to-severe pain and 13% experience daily pain (Randell et al., 2024). Pain typically affects multiple joints (Gowdie and Tse, 2012) and is strongly associated with reduced participation, limitations in daily functioning, and diminished quality of life (DeVine et al., 2011; Gudjonsdottir et al., 2024; Luque-Suarez et al., 2019). Chronic pain during childhood may also disrupt physical, functional, and psychosocial development, interfering with mobility, academic performance, and social interaction (Matcham et al., 2014). Neurobiological evidence indicates that persistent inflammatory pain may lead to alterations in cortical organization, attentional orienting to pain, and affective–motivational processing, potentially impacting descending modulation (Kaplan et al., 2022).

Patiens with JIA frequently exhibit elevated pain interference, disability, reduced physical activity, and altered somatosensory processing (Choong et al., 2024). Psychosocial factors, including anxiety, coping style, and family environment, further contribute to pain chronicity and interfere with sleep, academic functioning, and interpersonal relationships (Brandelli et al., 2023).

Persistent pain in JIA has also been linked to stress, depressive symptoms, low self-efficacy, and other cognitive-emotional vulnerabilities that may compromise neurodevelopment during adolescence (Alfven et al., 2019; Brandelli et al., 2023; Dudeney et al., 2024; Fair et al., 2019; Lozano-Meca et al., 2024; Miró et al., 2023; Somers et al., 2012; Tagliaferri et al., 2025). These findings align with contemporary neuromatrix and predictive-processing models, which emphasize interactions between nociception, central modulation, and cognitive–affective processes.

Current management of JIA remains primarily pharmacological, although the use of complementary rehabilitation strategies is increasing due to the multifactorial nature of the condition (Hashkes and Laxer, 2005; Ilowite, 2002; Wallace, 2006). While pharmacological interventions can alleviate symptoms, they do not fully address the cognitive, behavioral, and emotional factors that contribute to disability. Consequently, non-pharmacological approaches, including physiotherapy, exercise, and psychological therapies, have gained prominence due to their sustained benefits and lower risk of adverse effects (Stoll and Cron, 2014). In the field of rehabilitation, there is an increasing recognition of the importance of addressing both physical and neurocognitive factors that contribute to disability.

Among physiotherapeutic modalities, therapeutic exercise (TE) is one of the most effective interventions for improving joint mobility, strength, self-efficacy, emotional well-being, and cardiovascular fitness while decreasing pain and fatigue in pediatric and adult rheumatologic populations (Fransen et al., 2015; Hayden et al., 2021; Hu et al., 2021; Kisner et al., 2017; Sosa-Reina et al., 2017; Stephens et al., 2008). Therapeutic exercise can also modulate central pain mechanisms by enhancing endogenous inhibition, refining sensorimotor integration, and reducing threat appraisal.

Action observation therapy (AOT) is a therapeutic approach that involves observing goal-directed actions and subsequently executing them. This process relies on a mechanism of observation–execution matching, which involves the mirror neuron system and fronto-parietal visuomotor networks. This approach has demonstrated utility in neurorehabilitation and has been tested in populations with stroke, Parkinson’s disease, pediatric Cerebral Palsy, and orthopedic postoperative conditions (Buccino, 2014).

Active action observation (AAO), which involves the combination of intentional observation followed by immediate imitation, has been shown to produce greater corticospinal facilitation than passive observation or motor imagery (Roosink and Zijdewind, 2010). Neurophysiological evidence indicates that AAO enhances excitability of M1, Primary Motor Cortex, strengthens fronto-parietal connectivity, and supports motor learning.

Movement-representation strategies (AOT, motor imagery, mirror therapy) have shown efficacy in reducing pain in chronic musculoskeletal populations (Cuenca-Martínez et al., 2022), although effects vary across conditions. In the context of neck pain, a single AO (Action Observation) session has been shown to induce acute hypoalgesia comparable to observing natural scenes, underscoring the importance of extended dosing to influence fear, strength, and function (Al Shrbaji et al., 2023). From a biobehavioral perspective, AO may reduce fear–avoidance behaviors by weakening the learned association between movement and harm, potentially involving the amygdala and descending modulation systems (Raghava Neelapala and Shankaranarayana, 2020).

Chronic inflammation and persistent pain in JIA may be associated with altered sensorimotor processing and disrupted sensorimotor integration, leading to maladaptive cortical reorganization. This reorganization includes changes within somatosensory and motor networks that shape movement perception and control. Prolonged nociceptive input and repeated movement restriction may drive these changes by altering cortical representations and prediction processes related to joint movement and sensory feedback. Such alterations can reinforce protective movement patterns, fear of movement, and avoidance by biasing predictions about the threat value of movement-related sensations.

AAO may be uniquely suited to this context because it engages mirror neuron and visuomotor networks involved in action representation and motor prediction without requiring immediate physical execution. By repeatedly activating these networks in a low-load manner, AAO could support adaptive plasticity and recalibration of movement perception, potentially facilitating safer re-engagement with activity.

In pediatric populations, AO-based interventions have demonstrated feasibility and preliminary benefits, including improved bimanual function in children with hemiparesis, recruitment of premotor areas, and acceptability in home-based formats (Beani et al., 2020; Errante et al., 2024). Functional gains have also been described in adults following total knee arthroplasty and in Parkinson’s disease (Giorgi et al., 2018; Park et al., 2014). However, there is a lack of research on AAO in adolescents with JIA, despite the recognized relationship between chronic pain, fear of movement, and disrupted sensorimotor integration (De Oliveira et al., 2026; Kehl et al., 2024). AO-based interventions have shown promising results in pediatric neurorehabilitation (e.g., cerebral palsy) and have also been explored in adult rehabilitation and chronic pain contexts, where targeting motor representations may support functional recovery and symptom management. Despite this emerging evidence base, there is a complete lack of studies evaluating AAO/AOT approaches in pediatric rheumatology, including adolescents with JIA. This gap is notable given the high burden of pain-related disability and fear-driven movement avoidance in this population.

Furthermore, despite advances in pharmacological therapy, many adolescents with JIA continue to experience persistent pain, impaired motor function, and psychosocial difficulties that are inadequately addressed by standard care. There is an increasing demand for accessible, multimodal interventions that address the interaction between physical, cognitive, and emotional dimensions of disability. In this context, telerehabilitation (TR) has proven to be a cost-effective model that is adaptable, maintaining therapeutic intensity while reducing logistical and geographical barriers (Shambushankar et al., 2025). In addition, the available evidence supports its feasibility and positive effects on functional and psychosocial outcomes in both neurological and pediatric populations (Jiang et al., 2025). Therefore, implementing TR in combination with Active Action Observation allows safe and effective home-based delivery while enhancing adherence and autonomy, which are critical components in the daily management of adolescents with JIA.

Given the absence of prior studies exploring AAO in adolescents with JIA, this research adopts a prospective exploratory case series design. This descriptive and hypothesis-generating approach allows for detailed characterization of individual trajectories and interindividual variability, providing foundational data for future controlled trials aimed at establishing efficacy.

## 2 Methods

### 2.1 Study design

This study followed a prospective, exploratory case series design. It was conducted between July 2022 and January 2025. The methodological framework was based on the CARE (Case Report) guidelines for transparent reporting of case and case-series studies (Gagnier et al., 2013), complemented by selected elements of the STROBE statement to enhance clarity in the description of participants, variables, and data-collection procedures.

The protocol was reviewed and approved by the Ethics and Research Committee of Niño Jesús University Children’s Hospital (Madrid, Spain) in April 2022 (Approval No. R-0052/22). All study procedures were conducted in accordance with institutional and national standards for research involving human participants and adhered to the ethical principles outlined in the Declaration of Helsinki (World Medical Association, 2013).

This design was selected to allow for a comprehensive characterization of both individual and group trajectories following a home-based AAO TR program in adolescents diagnosed with JIA. Compared with controlled experimental designs, the case-series approach enables detailed documentation of response variability, feasibility, and preliminary therapeutic effects under real-world clinical conditions, providing foundational data for future controlled trials.

Participants were consecutively recruited from the pediatric rheumatology and rehabilitation units of the hospital. Prior to enrollment, adolescents and their parents or legal guardians received comprehensive verbal and written information regarding the study procedures, potential risks, and expected benefits. Written informed consent was obtained from parents or guardians, and assent was obtained from all adolescents prior to any assessment or intervention.

### 2.2 Participants

Participants were consecutively recruited from the Pediatric Rheumatology Units of Niño Jesús University Children’s Hospital and La Paz University Hospital (Madrid, Spain). Both hospitals are tertiary reference centers that specialize in pediatric rheumatology. Recruitment took place from July 2022 to November 2024. The screening and eligibility procedures were performed exclusively by a pediatric rheumatologist, who verified medical suitability using standardized diagnostic and clinical stability criteria.

Eligible participants were adolescents aged 11–17 years with a confirmed diagnosis of JIA according to the International League of Associations for Rheumatology (ILAR) criteria, under stable pharmacological treatment at the time of recruitment. Exclusion criteria included joint contractures or congenital anomalies that limit active movement; other musculoskeletal or cardiorespiratory conditions that could interfere with training; or an active inflammatory phase as determined by clinical and laboratory indicators.

All individuals who met these criteria were invited to participate voluntarily. Adolescents and their legal guardians provided written informed consent before inclusion. Prior to the AAO TR program, baseline demographic and clinical data were collected, including age, sex, disease duration, JIA subtype, pharmacological regimen, and functional status, were collected prior to the AAO TR program.

### 2.3 Study timeline

The study comprised 8 weeks of home-based intervention using a closed-access AAO program, delivered through a secure YouTube channel. Each week, participants accessed a new video containing the prescribed exercise sequence. During the intervention period, participants completed quantitative and qualitative assessments to evaluate changes across cognitive, emotional, motor, and somatosensory domains.

### 2.4 Quantitative assessments

Quantitative measurements were performed at three time points: baseline (T0), mid-intervention (T1, week 4), and post-intervention (T2, week 8).

### 2.5 Outcome measures

Emotional variables were assessed using the following tools: the PROMIS Pediatric Pain Interference Scale and the Stress Scale for Children (SSI-SM), the General Self-Efficacy Scale (GSES), and the Fear of Pain Questionnaire III (FOP-Q III) (Ceniza-Bordallo et al., 2022; Espejo et al., 2011; Sanjuán-Suárez et al., 2000; Solé et al., 2019).

Motor variables included performance on the Timed Up and Go test (TUG) and maximal grip strength of the dominant upper limb, assessed using a calibrated hand dynamometer and 6-min Walk Test (6MWT) (Mylius et al., 2016; Nicolini-Panisson and Donadio, 2013).

Somatosensory variables were measured using pressure pain thresholds (PPT) obtained with a digital algometer (Pain Test FPX 25 Wagner) over the cervical trapezius muscle of the dominant arm (Avellanal et al., 2020).

#### 2.5.1 Primary outcomes

The primary outcome was *pain interference*, quantified with the *PROMIS Pediatric Pain Interference Scale*, an 8-item instrument with four response options per item (Ceniza-Bordallo et al., 2022). The *minimally important difference* for this scale is 2.11 (SD = 0.59) (Thissen et al., 2016).

#### 2.5.2 Secondary outcomes

Secondary outcome measures included motor, psychological and somatosensory variables, which are presented below:

The Timed Up and Go (TUG) test is a standard evaluation tool used to assess functional mobility. The test involves the subject rising from a chair, walking a distance of 3 meters, and then returning to the chair. This test is used to assess functional mobility (Podsiadlo and Richardson, 1991). The minimal detectable change for this test is 1.26 (Martin et al., 2017).

Grip strength was measured by having the participants stand upright with their dominant arm alongside their body and squeeze the hand dynamometer as hard as they could (Wind et al., 2010). The smallest detectable difference is 2.16 (Gąsior et al., 2020).

The 6-min walk test (6MWT) is a tool used to evaluate a patient’s functional capacity at a submaximal level. The 6-min walk test (6MWT) was measured by the distance covered in 6 min (ATS Committee on Proficiency Standards for Clinical Pulmonary Function Laboratories, 2002). The minimal clinical important difference is 23 meters (Storm et al., 2020).

The Stress Manifestations Scale of the Student Stress Inventory (SSI-SM) is a 22-item scale that measures stress manifestations in the emotional, physiological, and behavioral domains (Espejo et al., 2011).

The general self-efficacy scale (GSES) is a 10-item instrument that measures an individual’s consistent sense of personal competence in dealing with a variety of stressful situations (Sanjuán-Suárez et al., 2000). The smallest detectable change is 4.33 (Ohno et al., 2017).

The Fear of pain questionnaire (FOP-Q III) is a 30-item scale designed to assess pain catastrophizing and anxiety sensitivity in relation to severe pain, minor pain, and medical pain (McNeil and Rainwater, 1998; Solé et al., 2019).

Cervical PPT was assessed with an algometer in the upper trapezius angle area (Avellanal et al., 2020; Winger et al., 2014).

### 2.6 Active action observation program

The AAO program was implemented over eight consecutive weeks in a TR format. This approach was designed to replicate the core components of Active Action Observation within a clinically meaningful rehabilitation context.

The AAO videos were streamed online via a private server. Access was restricted to authorized participants to protect confidentiality and maintain participant anonymity.

To ensure compliance with the established guidelines for weekly viewership, we utilized YouTube Analytics for objective monitoring of video visualizations from the private channel.

Each weekly module consisted of a pre-recorded video accessed remotely and followed in real time. YouTube videos were organized into the following categories:

1. Warm-up phase – focused on enhancing global joint mobility and developing dynamic flexibility
2. Main training phase – emphasizes multi-joint functional movements, integrating cardiovascular and resistance components to promote overall fitness.
3. Cool-down phase – involves diaphragmatic breathing and supine relaxation techniques to promote muscle recovery and relaxation.

Further details appear in Supplementary materials 1–3.

All videos were recorded from an allocentric (third-person) perspective, allowing clear visualization of whole-body kinematics. Participants were instructed to explicitly focus on movement trajectory, joint sequencing, tempo, and posture, enhancing the observation–execution matching mechanism. Immediate imitation of movements occurred after observation, or simultaneously when indicated.

The warm-up phase incorporated global joint mobility and dynamic flexibility exercises, progressing from the lower to the upper limbs (Supplementary material 1). This phase was designed to serve two primary purposes: to prepare the participants physiologically and to provide an initial opportunity to prime visuomotor attention and familiarize participants with the observation–execution sequence.

The main training phase comprised multi-joint functional movements integrating cardiovascular and resistance elements (Supplementary material 3). The exercises were intentionally selected to be ecologically relevant, easily reproducible at home, and designed to stimulate motor planning, intersegmental coordination, and sensorimotor integration. During this phase, the emphasis remained on observing with intention to imitate, followed by immediate execution to reinforce motor resonance.

The exercise program implemented in this study was specifically designed with the characteristics of the JIA population in mind. Program’s approach was methodical and incremental, with a duration of eight weeks. The selection of multi-joint functional exercises was based on the premise that adolescents with JIA engage in daily activities that require the use of multiple muscle groups, including movements similar to those performed in sports or games like basketball and soccer. Therefore, we selected exercises that not only involve functional movements but also simulate demands that adolescents may face in their daily lives. Furthermore, individualized demands were considered, and exercises were adapted to each participant’s capabilities. This adaptation was supported by the weekly qualitative assessments, which allowed us to monitor progress and make adjustments to the program as necessary.

The cool-down phase included diaphragmatic breathing and relaxation in a supine position (Supplementary material 2). Beyond physiological recovery, this phase was designed to down-regulate arousal and facilitate consolidation of motor and interoceptive signals after training.

The training frequency followed a progressive schedule, three sessions per week during weeks 1–3, four sessions per week during weeks 4–6, and five sessions per week during weeks 7–8. This schedule was designed to gradually increase exposure to the AAO stimulus while maintaining safety and adherence. Repetition counts were intentionally flexible, offering a target range to allow individualized pacing based on each adolescent’s functional status, fatigue, and daily symptom fluctuations.

Because AAO relies on active, attentive participation, so weekly videos were released only after completion of the qualitative follow-up session. This approach ensured that participants understood the instructions, reported no adverse responses to the previous session, and were ready to safely progress to the next training module. This monitoring strategy also reinforced adherence and optimized training load across the home-based TR program.

### 2.7 Weekly qualitative assessment

Patients participated in brief weekly online follow-up sessions via video call. A standardized set of questions were used to assess:

(1) The presence of discomfort or pain during the exercises was assessed by asking the following question: “Have you felt any discomfort while performing the exercises?”
(2) Delayed-onset muscle soreness (“Have you experienced muscle soreness after exercising?”)
(3) Overall satisfaction with the session (“Are you enjoying the treatment?”)
(4) Determination of specific exercises linked to discomfort, (“Is there any specific exercise that bothers you?”)
(5) Brief qualitative descriptors of pain or effort. (“Where does it hurt?,” “Could you describe what the discomfort feels like?”)

The online follow-ups consisted of semi-structured interviews in which participants were able to address both the questions asked as part of routine monitoring and any additional concerns that might arise in relation to the AAO protocol.

Two of these weekly assessments (weeks 4 and 8) were conducted in person, coinciding with the quantitative evaluations. This strategy, which involved a combination of monitoring methods, ensured the consistency of data collection and enhanced the clinical interpretation of the home-based intervention.

### 2.8 Statistical analysis

Given the prospective exploratory case-series design (*n* = 10), the statistical strategy was primarily descriptive and graphical. This strategy focused on individual trajectories and robust estimators of change rather than on formal hypothesis testing or *p*-values. All quantitative analyses were performed in R (R Foundation for Statistical Computing, Vienna, Austria).

For each outcome at the three assessment points, baseline (T0), week 4 (T1), and week 8 (T2), we computed conventional descriptive statistics including median, interquartile range (IQR), mean, standard deviation, minimum, and maximum.

To characterize within-participant evolution, we calculated absolute change scores (*Δ*) and percentage change (%Δ) for each interval (T0–T1, T1–T2, T0–T2). Percentage change was defined as follows:


$$\%\Delta =\frac{{\text{Score}}_{\text{later}}-{\text{Score}}_{\text{earlier}}}{{\text{Score}}_{\text{earlier}}}\times 100$$

For outcomes in which lower values represent clinical improvement (PROMIS Pain Interference, SSI-SM, FOPQ-III, TUG, cervical PPT), negative Δ and %Δ were interpreted as improvement. For outcomes where higher values indicate improvement (GSES, hand grip strength, 6-min walk test), positive Δ and %Δ were considered indicative of improvement. At the group level, we report the (interquartile range [IQR]) of the change in the primary outcome (Δ) and the percentage of the change in the primary outcome (Δ%), as well as the number of participants who improved or worsened from T0–T2.

Individual trajectories were visualized using spaghetti plots to highlight person-level change across the three time points. To present distributions while preserving individual variability, we generated combined graphical displays integrating boxplots (median and IQR), jittered individual data points, and connecting lines. Overall net change from baseline to post-intervention was illustrated using waterfall plots (individual Δ or %Δ) and forest-style dot plots of percentage change, which facilitate visual comparison of the magnitude and direction of change across participants and outcomes. All visualizations were generated using Python-based data visualization libraries (Matplotlib and Seaborn).

As a complement to these robust statistical methods, we computed Hodges–Lehmann (HL) estimators of median pairwise differences for each interval (T0–T1, T1–T2, T0–T2) and outcome. HL provides stable central estimates that are resistant to small-sample variability and outliers. It is important to note that Kendall’s coefficient of concordance (W) was calculated across T0, T1, and T2 for each variable to quantify the internal consistency of trajectory patterns over time. Importantly, Kendall’s W reflects the similarity in the shape of trajectories, not the magnitude or direction of improvement. Robust estimates are summarized in tabular information and were interpreted alongside descriptive statistics and graphical information rather than as inferential significance tests.

No parametric or rank-based hypothesis testing was planned in advance, in accordance with the exploratory and hypothesis-generating nature of this case series and the limited sample size. All participants provided complete data across the three assessments; therefore, no imputation procedures were required, and analyses were performed on a complete-case basis. Given the exploratory nature of the study and the limited sample size, it is essential to interpret the results descriptively rather than as confirmatory evidence.

## 3 Results

### 3.1 Sample characteristics

**Figure 1:**
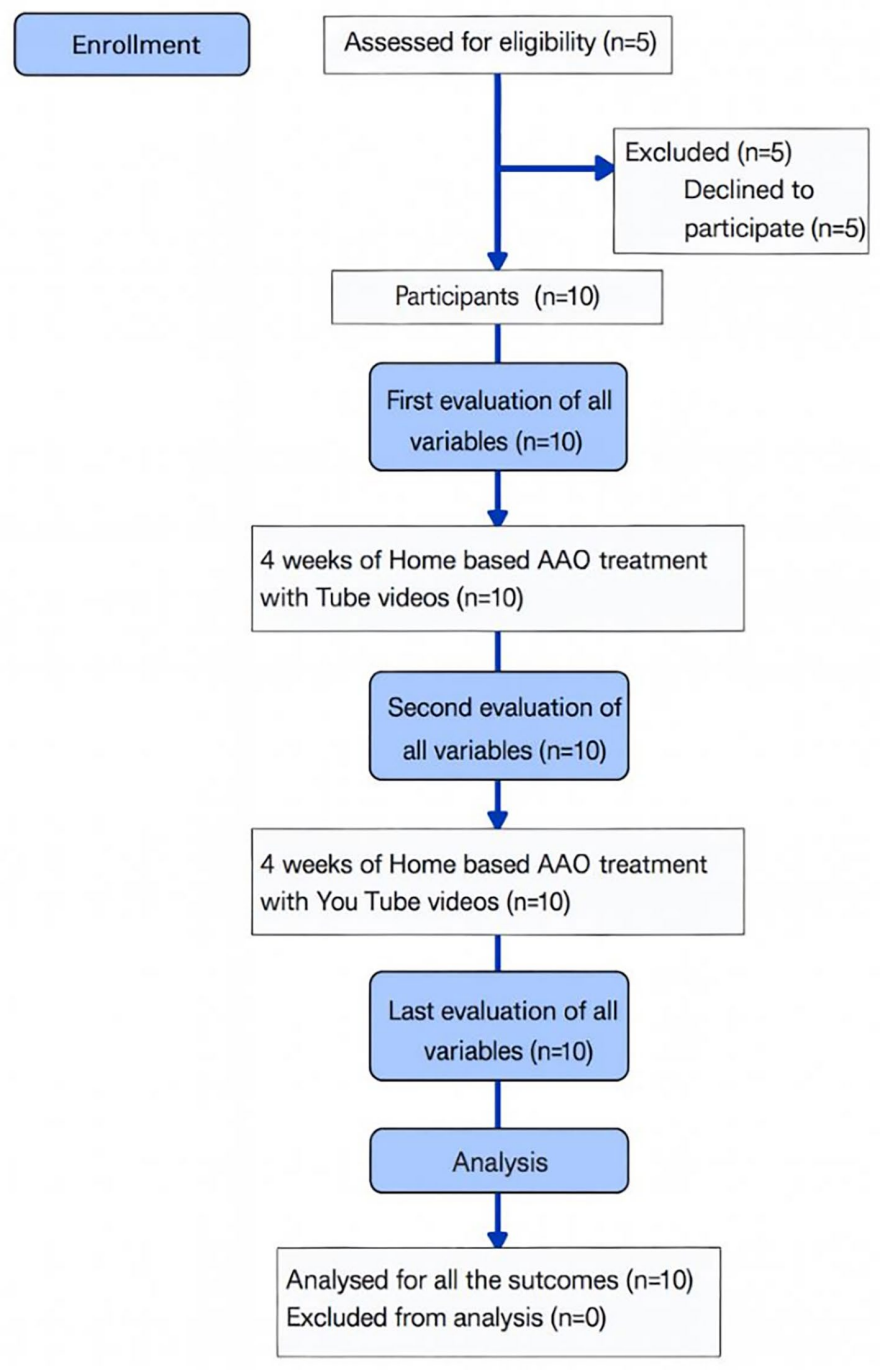
Patients’ flow diagram.

Ten adolescents with Juvenile Idiopathic Arthritis (JIA) participated in the study. The sample included 70% girls and was aged 11–17 years, with a mean age of 14.2 years. The body weight range was from 37 to 79 kilograms, and the height range was from 148 to 174 centimeters. The median disease duration was 71 months, with significant variability (interquartile range [IQR] = 89 months). The majority of participants were diagnosed with oligoarticular JIA (60%), followed by polyarticular (10%), psoriatic (10%), and enthesitis-related arthritis (10%). The baseline demographic and clinical characteristics reflected the heterogeneous presentation typical of JIA (Table 1). The patient flow diagram is presented in Figure 1.

**Table 1:** Participant characteristics.

Subject	Sex	Weight (kg)	Height (cm)	Age	JIA diagnosis time (months)	Type of JIA
1	Women	41	148	11	71	Polyarticular
2	Women	37	149	11	107	Oligoarticular
3	Women	42	152	12	115	Oligoarticular
4	Women	45	157	13	28	Oligoarticular
5	Women	52	160	14	7	Psoriatic
6	Men	79	174	15	117	Oligoarticular
7	Men	56	172	16	16	Oligoarticular
8	Women	64	172	16	36	Oligoarticular
9	Men	60	174	17	5	Enthesitis-related arthritis
10	Women	56	160	17	180	Oligoarticular

### 3.2 Qualitative results

During the eight-week intervention, reports of discomfort were infrequent, mild, and transient. No participant discontinued the program. Adherence and acceptability remained high throughout the study, as 100% of the participants who started the treatment completed it.

In week one, one participant with PwJIA reported transient lower back pain during kettlebell swings and three experienced muscle soreness; all participants but one reported enjoying the session. In week two, one participant reported experiencing knee pain, five participants reported experiencing muscle soreness, and all participants expressed a high level of satisfaction with the study. In week three, one participant experienced wrist discomfort during push-ups, and four participants reported muscle soreness. All participants stated they enjoyed the session. In week four, no participants reported pain; however, four participants experienced muscle soreness. Nine participants reported that the session was very enjoyable.

In week five, two adolescents experienced localized discomfort in the knee, hip, and gastrocnemius. Two others reported muscle soreness. Conversely, the session was well-received by the participants. In week six, three participants reported experiencing muscle soreness but not pain, and one participant reported a low level of enjoyment. In week 7, one participant reported experiencing abdominal pain and four participants reported muscle soreness. Two participants did not enjoy the session. In week 8, five participants experienced muscle soreness, and none reported pain. All participants but one enjoyed the session (Supplementary material 4).

### 3.3 Pain and psychological outcomes after intervention

**Figure 2:**
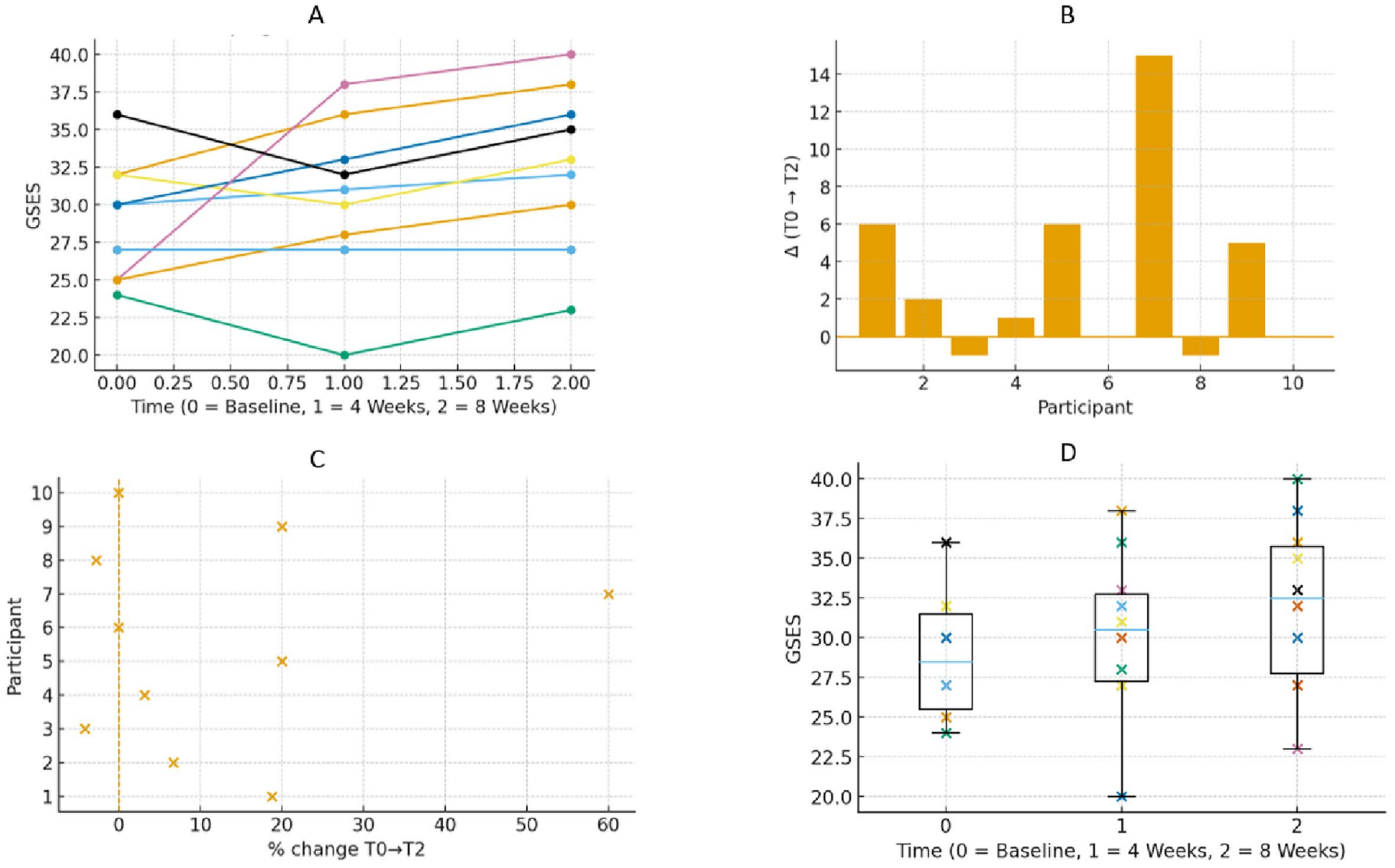
**(A)** Spaghetti plots shows trajectories of GSES for the different interferences of pain of the 10 participants. More punctuation is better. **(B)** Waterfall plots showing individual net change in GSES from baseline (TO) to post-intervention (T2), positive bars indicate improvement; negative bars indicate decline. Each bar corresponds to one participant. **(C)** Forest plot showing the percentage change in GSES from baseline (TO) to 8 weeks (T2) for each participant. The *x*-axis represents individual participants, and the *y*-axis represents their percentage change. More percentage is better improvement. **(D)** Box plot shows GSES at baseline (TO), 4 weeks (T1), and 8 weeks (T2) for all participants. Each boxplot shows the median, interquartile range, and individual data points for each time point. More punctuation is better.

Participants demonstrated a gradual decline in PROMIS Pain Interference over the course of the intervention. Specifically, medians decreased from 20 (IQR = 7.25) at baseline (T0) to 15 (IQR = 9) at week 4 (T1), and to 10 (IQR = 3.25) at week 8 (T2) (Table 2). Individual trajectories in pain interference were reduced between week 4 (T1) and week 8 (T2) (Figure 2). Median reductions were −6 points (−31.6%) from baseline (T0) to week 4 (T1) and −10 points (−42.1%) from baseline (T0) to week 8 (T2). Waterfall and percentage-change plots confirmed that most participants demonstrated clinically meaningful change, with several exceeding 40% (Figure 2). These results are supported by robust statistics: The HL estimators indicated reductions of −7 (from baseline (T0) to week 4 (T1)) and −10 (from baseline (T0) to week 8 (T2)), and Kendall’s W = 0.39 reflected moderate trajectory consistency. All 10 adolescents included in this study demonstrated a level of performance that met or exceeded the minimally important difference at least once (Table 2).

**Table 2:** Pain and psychological variables.

Patient	Measures	PROMIS	SSI-SM	GSES	FOP-Q III
1	Baseline	15	31	32	66
	4 weeks treatment	10	25	36	59
	Post treatment	10	26	38	56
2	Baseline	20	49	30	86
	4 weeks treatment	23	48	31	85
	Post treatment	17	38	32	65
3	Baseline	28	58	24	113
	4 weeks treatment	22	49	20	121
	Post treatment	13	45	23	127
4	Baseline	19	50	32	73
	4 weeks treatment	13	51	30	72
	Post treatment	11	47	33	60
5	Baseline	22	48	30	85
	4 weeks treatment	20	40	33	60
	Post treatment	10	29	36	47
6	Baseline	20	38	27	78
	4 weeks treatment	18	31	27	78
	Post treatment	10	33	27	61
7	Baseline	35	54	25	108
	4 weeks treatment	8	26	38	105
	Post treatment	9	25	40	109
8	Baseline	20	65	36	119
	4 weeks treatment	12	59	32	112
	Post treatment	26	54	35	120
9	Baseline	30	59	25	74
	4 weeks treatment	8	57	28	59
	Post treatment	9	54	30	55
10	Baseline	17	43	27	58
	4 weeks treatment	17	48	27	57
	Post treatment	8	40	27	55
Mean (SD)	Baseline	22.6 (±6.32)	49.5 (±10.12)	28.8 (± 3.9)	86.0 (± 20.7)
	4 weeks treatment	15.1 (±5.64)	43.4 (±12.32)	30.2 (± 5.1)	80.8 (± 24.1)
	Post treatment	12.3 (±5.45)	39.1 (±10.81)	32.1 (± 5.4)	75.5 (± 30.5)
Median	Baseline	20	49.5	28.5	81.5
	4 weeks treatment	15	48	30.5	75
	Post treatment	10	39	32.5	60.5
IQR	Baseline	7.25	13	5	23.5
	4 weeks treatment	9	17.25	5	35.5
	Post treatment	3.25	16.5	7	31.5

IQR, interquartile range.

**Figure 3:**
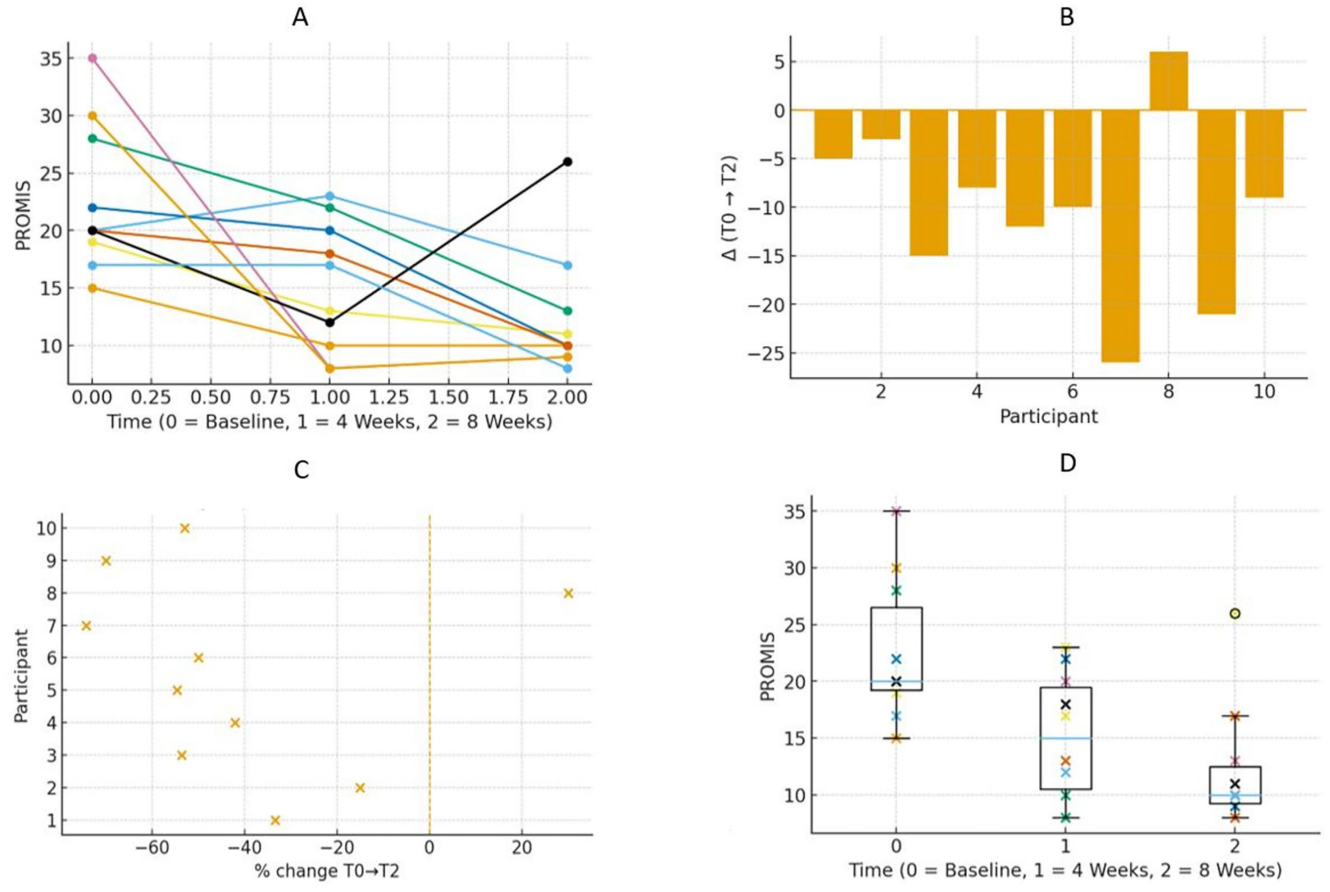
**(A)** Spaghetti plots shows trajectories of PROMIS for the different interferences of pain of the 10 participants. Less punctuation is better. **(B)** Waterfall plots showing individual net change in PROMIS from baseline (TO) to post-intervention (T2). Negative bars indicate improvement; positive bars indicate decline. Each bar corresponds to one participant. **(C)** Forest plot showing the percentage change in PROMIS from baseline (TO) to 8 weeks (T2) for each participant. The *x*-axis represents individual participants, and the *y*-axis represents their percentage change. Less percentage is better improvement. **(D)** Box plot shows PROMIS at baseline (TO), 4 weeks (T1), and 8 weeks (T2) for all participants. Each boxplot shows the median, interquartile range, and individual data points for each time point. Less punctuation is better.

SSI-SM scores showed a downward trend over time, with medians of 49.5 (IQR = 12.75) at baseline (T0), 48 at week 4 (T1), and 39 at week 8 (T2). The majority of participants exhibited a decline in scores and a reduction in dispersion at week 8 (T2) (Figure 3). According to the data, median reductions were −6 points (−10.6%) from baseline (T0) to week 4 (T1) and −11 points (−22.2%) from baseline (T0) to week 8 (T2). HL estimates supported these findings (−6 and −11, respectively). As shown in Table 2, Kendall’s W = 0.86, indicating a satisfactory level of coherence (Table 2).

**Figure 4:**
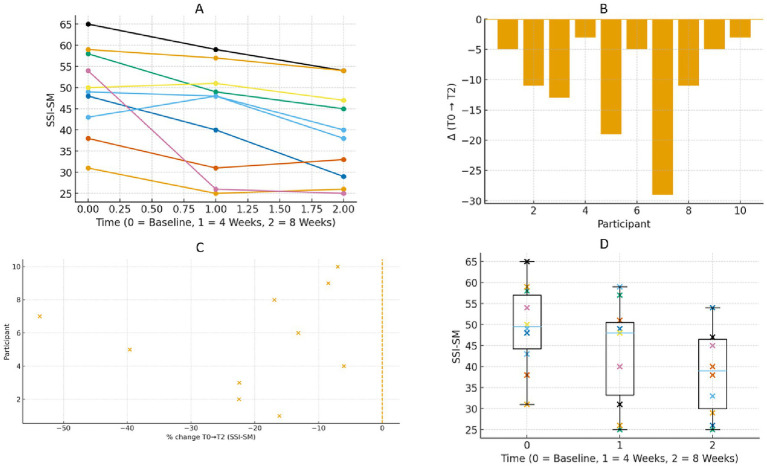
**(A)** Spaghetti plots shows trajectories of SSI-SM for the different interferences of pain of the 10 participants. Less punctuation is better. **(B)** Waterfall plots showing individual net change in SSI-SM from baseline (TO) to post-intervention (T2). Negative bars indicate improvement; positive bars indicate decline. Each bar corresponds to one participant. **(C)** Forest plot showing the percentage change in SSI-SM from baseline (TO) to 8 weeks (T2) for each participant. The *x*-axis represents individual participants, and the *y*-axis represents their percentage change. Less percentage is better improvement. **(D)** Box plot shows SSI-SM at baseline (TO), 4 weeks (T1), and 8 weeks (T2) for all participants. Each boxplot shows the median, interquartile range, and individual data points for each time point. Less punctuation is better.

GSES scores increased from 28.5 at baseline (T0) to 30.5 at week 4 (T1) and 32.5 at week 8 (T2) (Figure 4). Median change scores increased by two points (+10%) from baseline (T0) to week 4 (T1) and by 3 points (+18.8%) from baseline (T0) to week 8 (T2). Estimates for HL were +2 and +3, respectively. Kendall’s W value of 0.73 indicated moderate-to-high consistency across time. Four individuals exceeded the smallest detectable change (Table 2).

**Figure 5:**
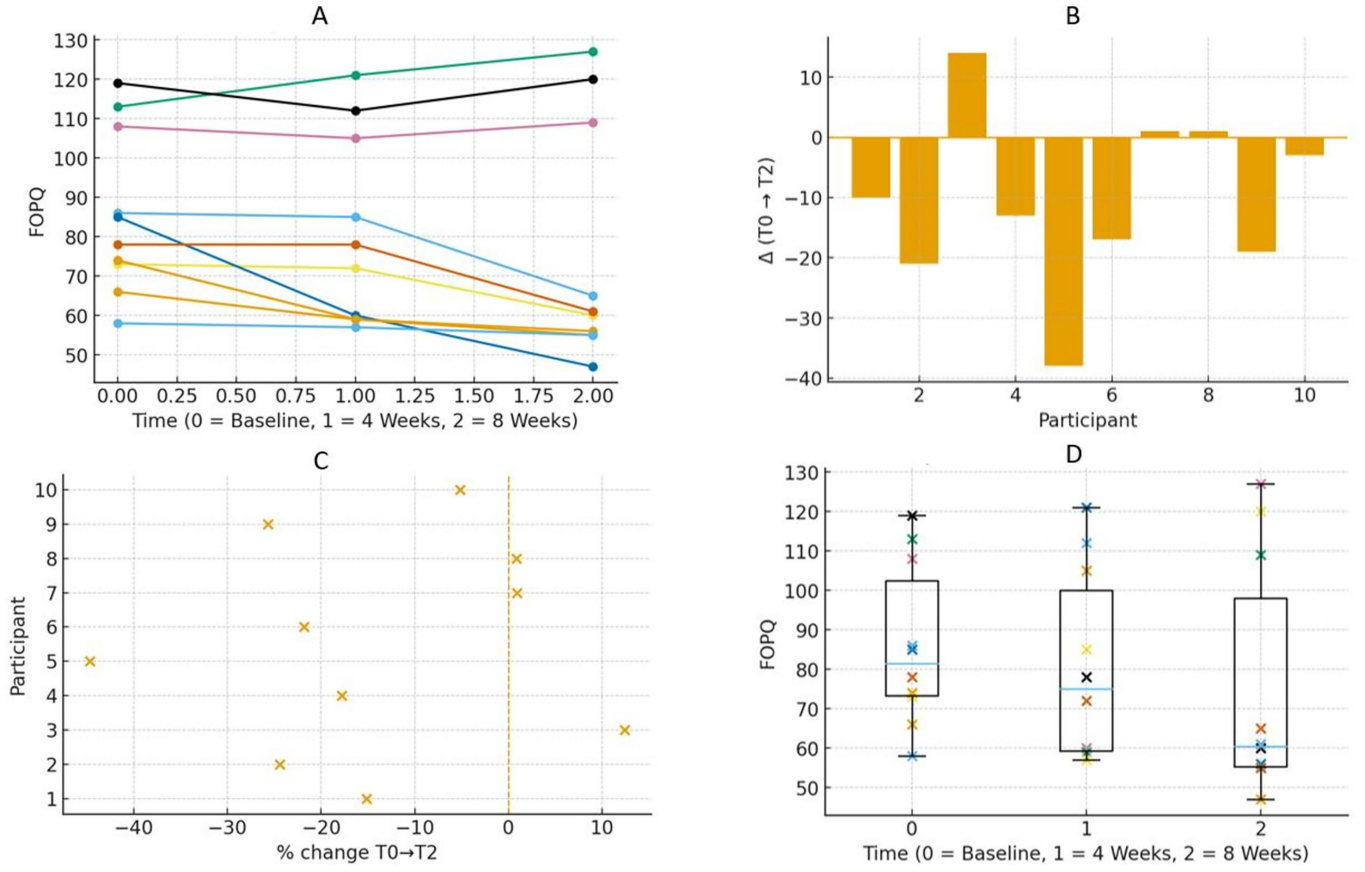
**(A)** Spaghetti plots shows trajectories of FOPQ-III for the different interferences of pain of the 10 participants. Less punctuation is better. **(B)** Waterfall plots showing individual net change in FOPQ-III from baseline (TO) to post-intervention (T2). Negative bars indicate improvement; positive bars indicate decline. Each bar corresponds to one participant. **(C)** Forest plot showing the percentage change in FOPQ-III from baseline (TO) to 8 weeks (T2) for each participant. The *x*-axis represents individual participants, and the *y*-axis represents their percentage change. Less percentage is better improvement. **(D)** Box plot shows FOPQ-III at baseline (TO), 4 weeks (T1), and 8 weeks (T2) for all participants. Each boxplot shows the median, interquartile range, and individual data points for each time point. Less punctuation is better.

As shown in Figure 5, FOPQ-III scores demonstrated a significant decrease over the eight-week period, with medians of 81.5 at baseline (T0), 75 at week 4 (T1), and 60.5 at week 8 (T2). At week 8 (T2), the narrowing of the distribution indicated a more uniform reduction in fear-related responses. The median percentage reductions were −10.6% (baseline (T0) to week 4 (T1)) and −25.7% (baseline (T0) to week 8 (T2)). The HL estimator indicated reductions of −7 and −15.5 points, respectively. Kendall’s W value of 0.90 demonstrated adequate consistency.

### 3.4 Motor outcomes

TUG performance showed changes across the study (Table 3). Specifically, the median decreased from 4.16 s at baseline (T0) to 3.75 s at week 4 (T1), remaining at 3.88 s at week 8 (T2) (Table 4). The findings showed a median reduction of −0.45 s (−11.18%) from baseline (T0) to week 4 (T1) and −0.69 s (−17%) from baseline (T0) to week 8 (T2). The HL estimator confirmed these findings (Table 5), showing a statistically significant decrease from baseline (T0) to week 8 (T2). Kendall’s W (0.91) also demonstrated adequate consistency, indicating the reliability of the data. One adolescent exceeded the minimal detectable change threshold (Table 3).

**Table 3:** Motor and somatosensorial variables.

Patient	Measures	TUG	Hand grip	6-minute walk test	Cervical PPT
1	Baseline	4.4	14	620	3.2
	4 weeks treatment	4.22	15.5	660	3.28
	Post treatment	3.89	15	700	4.45
2	Baseline	4.08	18.66	824	3.11
	4 weeks treatment	3.81	20	786	2.45
	Post treatment	3.93	20	775	2.68
3	Baseline	4.58	16	744	3.8
	4 weeks treatment	3.98	18	718	2.31
	Post treatment	4.38	13.66	672	1.79
4	Baseline	4.2	24.33	645	2.1
	4 weeks treatment	3.66	24.33	645	2.16
	Post treatment	3.88	21	560	2.67
5	Baseline	3.81	20	755	2.85
	4 weeks treatment	3.26	21.3	755	3.09
	Post treatment	3.4	18	770	3.12
6	Baseline	3.7	33.66	730	4.52
	4 weeks treatment	3.23	27.33	750	3.69
	Post treatment	2.78	24.66	824	4.88
7	Baseline	5.55	34.33	620	5.32
	4 weeks treatment	4.78	38.33	910	2.91
	Post treatment	4.1	36.66	970	3.89
8	Baseline	4.39	26	725	3.12
	4 weeks treatment	4.33	27	708	3.64
	Post treatment	4.25	22.66	730	1.91
9	Baseline	3.52	43.33	792	3.74
	4 weeks treatment	2.91	42.66	795	2.99
	Post treatment	3.03	46	744	2.27
10	Baseline	4.13	22.33	744	3.35
	4 weeks treatment	3.7	23.66	725	2.85
	Post treatment	3.44	27	756	2.69
Mean	Baseline	4.23 (±0.56)	25.26 (±9.26)	719.9 (± 70.0)	3.51 (±0.9)
	4 weeks treatment	3.78 (±0.56)	25.81 (±8.66)	745.2 (± 75.5)	2.93 (±0.52)
	Post treatment	3.70 (±0.52)	24.46 (±10.01)	750.1 (± 105.4)	3.03 (±1.05)
Median	Baseline	4.16	23.33	737	3.27
	4 weeks treatment	3.75	23.99	737.5	2.95
	Post treatment	3.88	21.83	750	2.68
IQR	Baseline	0.52	12.74	64.5	0.67
	4 weeks treatment	0.8	6.99	57.5	0.68
	Post treatment	0.64	7.91	57.5	1.32

IQR, interquartilic range.

**Table 4:** Descriptive statistics for motor and somatosensory outcomes.

Variable	T0 Median (IQR)	T1 Median (IQR)	T2 Median (IQR)	Interpretation
TUG (s)	4.16 (0.52)	3.75 (0.80)	3.88 (0.64)	Lower values indicate better performance
Hand grip (kg)	23.33 (12.74)	23.99 (6.99)	21.83 (7.91)	Higher values indicate better performance
6MWT (m)	737 (64.5)	737.5 (57.5)	750 (57.5)	Higher values indicate better performance
Cervical PPT	3.27 (0.67)	2.95 (0.68)	2.68 (1.32)	Higher values indicate better performance

Descriptive statistics include median and interquartile range (IQR).

**Table 5:** Change scores and statistics.

Variable	Δ Median (IQR)	%Δ Median (IQR)	Improved	Worsened	HL T0–T2	Kendall W
TUG	−0.45 (0.41)	−11.18% (10.83)	10	0	−0.435	0.91
Hand grip	−0.50 (5.16)	−1.92% (20.53)	5	5	−0.830	0.96
6MWT	+8.50 (112.50)	+1.15% (16.19)	6	4	+23	0.67
PPT	−0.54 (1.71)	−16.76% (44.90)	4	6	−0.525	0.54

HL, Hodges–Lehmann estimator. Kendall W indicates within-subject concordance. For TUG, lower values indicate improvement; for Hand Grip, 6MWT, and PPT, higher values indicate improvement. Thus, for PPT, negative Δ and %Δ reflect worsening (reduced pressure pain thresholds), whereas positive values indicate improvement.

In regard to handgrip strength, it is important to note the results appear to be heterogeneous. Specifically, some participants showed improvements by week 4 (T1), with group-level medians shifting from 23.33 kg at baseline (T0) to 23.99 kg in week 4 (T1) and then decreasing to 21.83 kg in week 8 (T2), as shown in Table 4. The percentage changes for each individual ranged widely, from a low of −26.7% to a high of +20.9%. The HL estimator revealed a modest overall decline of −0.83 from baseline (T0) to week 8 (T2). Meanwhile, Kendall’s W value of 0.96 indicated adequate consistency (Table 5). Two participants met the smallest detectable change threshold (Table 3).

In the 6 MWT, walking distance showed a slight increase at the group level, with median walking distances of 737 m at baseline (T0), 737.5 meters at week 4 (T1), and 750 meters at week 8 (T2), as shown in Table 4. Individual responses exhibited significant variation, with percentage changes ranging from −13 to +56%. The HL estimator indicated a robust overall improvement of +23 m (baseline (T0) to week 8 (T2)). Furthermore, Kendall’s W value of 0.67 suggested moderate-to-high consistency in aerobic capacity changes across time, as shown in Table 5. Four individuals demonstrated a response that exceeded the minimal clinically important difference (Table 3).

### 3.5 Somatosensory outcomes

The PPT values showed a progressive decrease, with medians of 3.27 kg/cm^2^ at baseline (T0), 2.95 in week 4 (T1), and 2.68 in week 8 (T2) (Table 1). Percentage changes ranged widely across participants (−52 to +39%) (Table 3). According to the estimates provided by HL, there has been a modest overall reduction of approximately −0.48 and −0.53 (Table 5). Kendall’s W was 0.54, which indicated moderate coherence. This suggests that there is consistency in the individual trajectories, but the direction of change is not uniform (Table 5). Given that lower PPT values indicate increased pain sensitivity, these reductions reflect a deterioration in somatosensory responses in a subset of the sample.

## 4 Discussion

This prospective, exploratory case series examined the feasibility, acceptability, and preliminary clinical effects of an eight-week home-based AAO TR program in adolescents with JIA. To the best of our knowledge, this is the first study to apply AAO in this population. Overall, the results indicate that AAO delivered through a progressively structured video-based program is safe, well accepted, and associated with meaningful changes in pain interference, stress, self-efficacy, and fear of pain/movement. However, motor and somatosensory outcomes showed more heterogeneous trajectories.

### 4.1 Summary of key findings

During the intervention period, participants demonstrated a marked decrease in pain interference, with median improvements surpassing 40% from baseline to week 8. Stress levels decreased, general self-efficacy increased, and fear-related responses on the FOPQ-III declined substantially. These changes were supported by solid estimates of central tendency (Hodges–Lehmann) and substantial agreement across the three assessments (Kendall’s W), indicating coherent individual trajectories despite the small sample size.

In terms of motor performance, functional mobility as measured by TUG, demonstrated consistent improvement. In contrast, the 6MWT exhibited modest gains and significant interindividual variability. Handgrip strength exhibited a biphasic pattern, with improvements observed in some adolescents and reductions in others. Somatosensory responses assessed by cervical PPT were particularly heterogeneous, with some participants demonstrating decreased thresholds (greater sensitivity) and others showing stability or improvement. The qualitative weekly monitoring revealed high adherence, good tolerability, and positive treatment perceptions, with only mild and transient discomfort reported and no withdrawals.

### 4.2 Integration with evidence on action observation and motor representation techniques

The progressive reductions observed in pain interference and stress are consistent with existing evidence on movement representation techniques, such as action observation, motor imagery, and mirror therapy. These techniques have been shown to reduce pain and improve functional outcomes in adults with chronic musculoskeletal conditions (Cuenca-Martínez et al., 2022). These interventions may contribute to changes in cortical excitability and descending modulation, and have been proposed to influence maladaptive pain-related cognitions (Larsen et al., 2019).

In pediatric populations with chronic pain, including JIA, high levels of pain interference and disability are closely associated with anxiety, stress, reduced self-efficacy, and other psychosocial vulnerabilities that can impair school functioning, social participation, and emotional development (Brandelli et al., 2023; Gudjonsdottir et al., 2024; Miró et al., 2023; Tagliaferri et al., 2025). The significant increases in self-efficacy seen in this study are therefore of clinical relevance. Greater perceived competence has been associated with better adjustment, lower disability, and higher adherence to rehabilitation in adolescents with chronic pain (Black et al., 2025).

Research has identified fear of movement and pain-related avoidance as significant contributors to disability in pediatric musculoskeletal conditions, including JIA (Lozano-Meca et al., 2024; Luque-Suarez et al., 2019). From a biobehavioral perspective, AAO has the potential to mitigate these fears by providing graded observational exposure to functional movements performed without apparent harm. Studies have shown that observing goal-directed actions executed successfully and then imitating them under controlled conditions can help weaken the learned association between movement and danger. This response is in line with fear-avoidance models and observational learning frameworks (Cameron et al., 2016; Fryling et al., 2011; Raghava Neelapala and Shankaranarayana, 2020). The significant decreases in FOPQ-III scores over the eight-week period are consistent with this mechanism and suggest that AAO may serve as a form of non-threatening, motor-based exposure.

The neurophysiological rationale underlying these effects is supported by experimental studies on the mirror neuron system and fronto-parietal visuomotor networks. Action observation engages premotor, parietal, and primary motor areas, facilitating motor planning and execution, particularly when observation is active and immediately followed by imitation (Buccino et al., 2001).

Although AAO has not yet been widely studied, other closely related approaches such as AO have been investigated in pain populations. Overall, AO based training shows promise as a therapeutic approach for individuals experiencing pain. By engaging sensorimotor networks through the observation of goal-directed movements, often combined with subsequent execution, it may help reduce fear-related responses to movement, support motor relearning, and improve functional performance, particularly in conditions where pain is closely linked to avoidance and altered movement patterns. However, the evidence is not uniform across all pain presentations: results seem less consistent in populations such as individuals with phantom limb pain or post-stroke pain, where responses to observation-based interventions have been more variable (Cuenca-Martínez et al., 2022).

In JIA, where disuse, pain, and altered proprioception may disrupt normal neuromotor development, AAO offers a way to re-engage these networks while reducing physical and emotional demands. Evidence from pediatric populations with cerebral palsy has shown that AO-based protocols can improve bimanual performance and premotor activation and can be delivered in acceptable home-based formats (Beani et al., 2020; Errante et al., 2024; Fierro-Marrero et al., 2025; Ortega-Martínez et al., 2023). Functional benefits have also been reported in adults with Parkinson’s disease and after total knee arthroplasty (Giorgi et al., 2018; Mezzarobba et al., 2024; Park et al., 2014; Villafañe et al., 2017). The present findings extend this line of research to adolescents with JIA, suggesting that AAO may help integrate motor, cognitive, and emotional components of rehabilitation.

### 4.3 Potential mechanisms of change

The improvements observed may be indicative of interacting top-down and bottom-up processes. Studies have shown that engaging in structured and achievable movement tasks can help reduce threat appraisal and increase perceived control, which supports more adaptive interpretations of bodily sensations. This interpretation aligns with the observed reductions in stress and increases in self-efficacy, which are factors known to influence symptom management and functional participation in pediatric rheumatologic conditions (Abele and Spurk, 2009; Somers et al., 2012).

At a sensorimotor level, repeated cycles of action observation and movement execution may strengthen visuomotor coupling and enhance confidence in movement. Despite the absence of significant changes in strength or endurance, these processes have the potential to enhance functional performance in a more efficient or economical manner. This aligns with the observed improvements detected in TUG times, as reported by Keramiotou et al. (2020).

These mechanisms align with contemporary biobehavioral models of chronic pain, in which the pain experience emerges from dynamic interactions among sensory input, central modulation, cognitive appraisal, emotional responses, and overt behavior. AAO can be conceptualized as a multimodal stimulus influencing multiple components of this system simultaneously (Vlaeyen et al., 2016).

Given the exploratory nature of this case series, the mechanisms discussed should be interpreted as hypothesis-generating interpretations based on our observations; future studies using controlled designs and neurophysiological measures are needed to directly test these proposed mechanisms.

### 4.4 Heterogeneity of motor and somatosensory outcomes

The differences observed in grip strength, the 6MWT, and PPT responses require careful analysis. JIA is a clinically heterogeneous condition with substantial variability in joint involvement, systemic features, and disease activity. Therefore, differential responses to an eight-week program are expected.

For grip strength, fluctuations in wrist or finger synovitis, fatigue, maturation, or medication changes may contribute to variability, but there is no clear evidence of improvements. Although Kendall’s W indicated internally consistent trajectories for each participant, this reflects stability of individual patterns rather than agreement in the direction of change. Given that the consistency analysis is derived from our proprietary dataset, external citations are not required.

The modest median improvement in the 6MWT, coupled with significant individual variability, is likely attributable to differences in baseline fitness, motivation, symptom fluctuation, and the recognized sensitivity of submaximal walking tests to multiple physical and psychosocial influences in adolescents.

The PPT findings were the most varied. While group medians showed decreased thresholds, indicating heightened sensitivity, individual responses ranged from substantial decreases to meaningful increases. Pain sensitivity in JIA is influenced by peripheral inflammation and central mechanisms such as sensitization, descending modulation, sleep quality, and emotional distress (Huber et al., 2022; Karshikoff et al., 2016; Liew et al., 2022).

The somatosensory results observed in this study appear to be consistent with the existing literature, in which various authors have reported sensory alterations in PwJIA, including in areas without inflammation, during non-active phases of the disease, and even in adults with a long disease duration (Arnstad et al., 2020; Cornelissen et al., 2014; Leegaard et al., 2013).

Patients with JIA, given the long-term and recurrent nature of the disease, may develop more pronounced sensory alterations than individuals with acute conditions. This prolonged exposure to pain and inflammation may have a significant impact on central nervous system processing, potentially requiring more comprehensive and sustained management. Consequently, somatosensory abnormalities may require a longer intervention period to elicit meaningful neuroplastic changes (Sturgeon et al., 2024).

It is plausible that AAO primarily influenced cognitive-emotional and functional domains during the eight-week window, whereas somatosensory modulation may follow a slower or more variable trajectory. These results underscore the fact that improvements in subjective and functional outcomes do not necessarily parallel changes in experimental pain measures; moreover, in pediatric and adolescent populations, pressure pain thresholds have been reported to show weak or no association with improvements in functional capacity (Lim et al., 2017).

### 4.5 Feasibility and acceptability of home-based AAO with clinical implications

One of the study’s strengths is the demonstration that a structured AAO program can be safely and feasibly delivered at home to adolescents with JIA using simple digital infrastructure. Initial findings indicate that AAO holds promise as an effective adjunctive strategy in the multidisciplinary management of adolescents with JIA. The results of the study indicate that AAO may be particularly useful for targeting pain interference, stress, self-efficacy, and fear-avoidance, domains that are often insufficiently addressed by pharmacological treatment alone. The modest but consistent improvements in functional mobility reinforce its potential role as a preparatory or complementary intervention to more intensive exercise-based programs.

Adherence was high, discomfort was mild and transient, and participants generally reported enjoying the sessions. These findings align with previous research showing that TR is a cost-effective and adaptable model that can reduce logistical barriers while maintaining therapeutic intensity in pediatric populations with rheumatologic conditions (Armbrust et al., 2017; Seron et al., 2021). For adolescents who often face competing school demands, transportation challenges, or fatigue, home-based rehabilitation may be particularly advantageous. The TR format has the potential to enhance accessibility and promote continuity of care, particularly for families residing in distant locations or encountering financial limitations. Furthermore, the program emphasizes autonomy and active participation, both of which are critical for successful transition from pediatric to adult care in chronic rheumatologic conditions.

The viability of digitally facilitated rehabilitation is further substantiated by the findings of Butler et al. (2024). These researchers examined a smartwatch- and app-based system for remote monitoring of pain, medication adherence, and physical activity in young people with JIA (Butler et al., 2024). Their findings indicate that real-time data collection was both acceptable and technically feasible in this age group. The study highlights the opportunities as well as the behavioral challenges inherent in remote monitoring. These findings align with our own experience, indicating that effective remote physiotherapy services require simple interfaces, flexible scheduling, and proactive engagement strategies to ensure sustained participation over time.

Additionally, the weekly qualitative check-ins in our study appear to have played a meaningful role in maintaining engagement, monitoring adverse events, and tailoring progression. This combination of structured digital content with brief synchronous supervision may represent a pragmatic model for implementing AAO in clinical practice, preserving the advantages of home-based delivery while ensuring adequate therapeutic oversight.

Our findings align with those of a recent synthesis by Ren et al. (2025), who reported small-to-moderate improvements in pain and physical activity in JIA populations receiving digital interventions compared with usual care (Ren et al., 2025). Their meta-analysis suggests that digital self-management tools and remotely delivered rehabilitation programs can produce clinically relevant changes, particularly when they incorporate interactive education, symptom tracking, and behavioral coaching. In this context, our study builds on existing evidence by demonstrating that an action-observation–based TR protocol is also practical and linked to meaningful improvements in pain interference and functional outcomes, even within a relatively short eight-week intervention period.

### 4.6 Limitations

It is important to acknowledge the limitations of this study when interpreting the results. First, the sample was small and heterogeneous with respect to JIA subtypes and disease duration, which limits generalizability and increases the influence of individual variability. Second, the lack of a control group limits the ability to make causal inferences. The observed improvements may be partially attributable to non-specific factors such as attention, expectations, or natural fluctuations in disease activity. The adherence to the intervention was assessed exclusively through self-report measures. While this approach is common in feasibility studies, it is subject to reporting bias and limits the objectivity of adherence estimates. Future research should incorporate objective or multimodal adherence measures, such as device-based monitoring or therapist logs, to enhance the study’s reliability and validity. Additionally, the lack of blinding in outcome assessments introduces the potential for expectancy or observer bias, which can compromise the objectivity and integrity of the results. Although this limitation is inherent to many early-phase and feasibility studies, blinded outcome assessment should be prioritized in subsequent controlled trials to strengthen internal validity. Furthermore, the study’s design entailed repeated and relatively frequent assessments, which might have introduced measurement reactivity. This is a potential concern where participants’ responses or behaviors could have been influenced by ongoing monitoring. This potential reactivity may have contributed to the observed changes over time and should be considered when interpreting longitudinal trends.

The intervention spanned 8 weeks, and longer-term studies are necessary to ascertain the longevity of the effects and whether more pronounced motor or somatosensory changes emerge over time. Fourth, PPT was assessed at a single cervical site, which may not fully capture generalized changes in pain sensitivity. Additionally, while the intervention was outlined at a conceptual level, the procedural details provided are limited. This limitation hinders the full replicability of the results, a concern that should be addressed in future studies through more detailed reporting of intervention components, dose, progression, and delivery procedures. Lastly, the absence of neurophysiological measures precludes any definitive conclusions regarding the underlying mechanisms remain speculative.

### 4.7 Future directions

Future research should build on these exploratory results by conducting randomized controlled trials. These trials should compare AAO plus usual care with usual care alone, as well as with traditional exercise-based interventions. Larger samples would allow exploration of moderators and mediators of response, such as JIA subtype, baseline fear of movement, or self-efficacy. Incorporating neurophysiological measures such as EEG, fMRI, or TMS could help clarify the neural correlates of AAO in this population and test hypothesized mechanisms related to mirror neuron and visuomotor network activation. It would also be informative to examine different AAO “doses,” levels of task complexity, and combinations with other biobehavioral strategies, including therapeutic education or cognitive-behavioral interventions. Finally, the development of adaptive digital platforms capable of tailoring intensity and content to individual performance may optimize engagement and outcomes.

## 5 Conclusion

In summary, this exploratory case series demonstrated the feasibility and acceptability of home-based Active Action Observation delivered through TR for adolescents with Juvenile Idiopathic Arthritis. Across the study period, participants exhibited positive trends in psychological outcomes (including pain interference, stress-related symptoms, self-efficacy, and fear of pain/movement), accompanied by modest changes in functional performance. Somatosensory outcomes demonstrated mixed and heterogeneous patterns. These findings must be interpreted with caution. The study was exploratory in nature, involved a small sample size, and lacked a control group, precluding causal inference regarding intervention effects. The findings, which were generated to generate hypotheses, collectively provide substantial support for the necessity of a randomized controlled trial. Such a trial would allow for the rigorous evaluation of the efficacy of Active Action Observation. Additionally, it would facilitate the elucidation of its underlying mechanisms of action in adolescents with Juvenile Idiopathic Arthritis.

## Data Availability

The original contributions presented in the study are included in the article/Supplementary material, further inquiries can be directed to the corresponding author.
