# Supplementary material for: Effects of active action observation on cognitive, emotional, motor, and somatosensory outcomes in adolescents with juvenile idiopathic arthritis: a prospective exploratory case series
Source: Front Hum Neurosci. 2026 Feb 27;20:1766070. doi: 10.3389/fnhum.2026.1766070 (PMC12982409; doi:10.3389/fnhum.2026.1766070)
Supplement: Supplementary file 1 [file Supplementary_file_1.zip › Supplementary Material/Supplementary Material 1.docx]

WARM UP

| Exercise | Explanation |
| --- | --- |
| Circles with ankles / Change direction | With the tip of the toe on the ground, active ankle circles are performed; after 10 seconds, the direction of the circle is changed. |
| Circles with knees / Change direction | Both knees together make circles for 10 seconds, then the direction of the circles changes. |
| Hip circles/ Change direction | With both hands resting on our hips, we will make circles for 10 seconds, then change direction. |
| Touch the tips of our toes | We bend forward, trying to touch the tips of our toes with our hands. |
| Lumbar extension | With hands resting on the hips, we try to do lumbar extension as much as possible. |
| Spinal rotation | Hand on hand at chest level with arm extended, we rotate to both sides. |
| Spinal lateral flexion/ Change direction | With one hand resting on the hip and the other hand passing over the head, we perform spinal lateral flexions then we change sides. |
| Cervical flexo-extension | Cervical maximum flexion and extensions are performed. |
| Cervical rotation | We rotate the neck to both sides, looking at one shoulder and then the other. |
| Shoulder rotation | Shoulder circles are performed first in one direction and then in the other. |
| Elbow flexo-extension | An elbow flexion-extension is performed. |
| Wrist circles | Wrist circles are performed in one direction, then the direction is changed. |
| Open the palm of the hand and close the fist | We open the fingers and then clench the fist tightly. |

All the exercises were performed for a period of 10 to 20 seconds, and all will be done standing.
