# Supplementary material for: Effects of active action observation on cognitive, emotional, motor, and somatosensory outcomes in adolescents with juvenile idiopathic arthritis: a prospective exploratory case series
Source: Front Hum Neurosci. 2026 Feb 27;20:1766070. doi: 10.3389/fnhum.2026.1766070 (PMC12982409; doi:10.3389/fnhum.2026.1766070)
Supplement: Supplementary file 1 [file Supplementary_file_1.zip › Supplementary Material/Supplementary Material 2.docx]

| WEEK 1 (REPEAT 3 DAYS THE TRAINING) | | |
| --- | --- | --- |
| WARM UP | | |
| Knees up | 2 SERIES | 30 SECONDS |
| Squats with seat | 2 SERIES | 10-12 REPS |
| Bench press (5kg) | 2 SERIES | 10-12 REPS |
| Abbs with ball | 2 SERIES | 10-12 REPS |
| Ketelbell swing (5kg) | 2 SERIES | 10-12 REPS |
| CALM DOWN | | |

| WEEK 2 (REPEAT 3 DAYS THE TRAINING) | | |
| --- | --- | --- |
| WARM UP | | |
| Star jump | 2 SERIES | 10-12 REPS |
| Sumo squat | 2 SERIES | 10-12 REPS |
| Stand french press | 2 SERIES | 10-12 REPS |
| Leg raises | 2 SERIES | 10-12 REPS |
| Lunges with ball | 2 SERIES | 10-12 REPS |
| Mountain climbers | 2 SERIES | 30 SECONDS |
| CALM DOWN | | |

| WEEK 3 (REPEAT 3 DAYS THE TRAINING) | | |
| --- | --- | --- |
| WARM UP | | |
| Jump rope | 2 SERIES | 15-20 REPS |
| Lunges | 2 SERIES | 15-20 REPS |
| Push-ups with chair | 2 SERIES | 15-20 REPS |
| Plank | 2 SERIES | 20 SECONDS |
| Boxing | 2 SERIES | 15-20 REPS |
| Deadlift (5kg) | 2 SERIES | 40 SECONDS |
| CALM DOWN | | |

| WEEK 4 (REPEAT 4 DAYS THE TRAINING) | | |
| --- | --- | --- |
| WARM UP | | |
| Star jump | 2 SERIES | 15-20 |
| Jumping squat | 2 SERIES | 15-20 REPS |
| Curl biceps (5 kg) | 2 SERIES | 15-20 REPS |
| Leg raises | 2 SERIES | 15-20 REPS |
| Touching colateral toe | 2 SERIES | 15-20 REPS |
| Mountain climbers | 2 SERIES | 40 SECONDS |
| CALM DOWN | | |

| WEEK 5 (REPEAT 4 DAYS THE TRAINING) | | |
| --- | --- | --- |
| WARM UP | | |
| Butt kicks | 3 SERIES | 15-20 REPS |
| Jumping sumo squat | 3 SERIES | 15-20 REPS |
| Bench press (5kg) | 3 SERIES | 15-20 REPS |
| Abs with ball | 3 SERIES | 30 SECONDS |
| Kettkebell swing | 3 SERIES | 15-20 REPS |
| Single-leg glute bridge | 3 SERIES | 40 SECONDS |
| Leg raise touching the toe | 3 SERIES | 10-12 REPS |
| CALM DOWN | | |

| WEEK 6 (REPEAT 4 DAYS THE TRAINING) | | |
| --- | --- | --- |
| WARM UP | | |
| Star jumping | 3 | 15-20 REPS |
| Push up in step | 3 | 15-20 REPS |
| Jumping lunge | 3 | 15-20 REPS |
| Plank | 3 | 30 SECONDS |
| Lunge with overhead press (5 kg) | 3 | 15-20 REPS |
| Boxing | 3 | 40 SECONDS |
| Burpee | 3 | 10-12 REPS |
| CALM DOWN | | |

| WEEK 7 (REPEAT 5 DAYS THE TRAINING) | | |
| --- | --- | --- |
| WARM UP | | |
| Butt kicks | 3 SERIES | 15-20 REPS |
| Jumping squat | 3 SERIES | 15-20 REPS |
| Bench press (5 kg) | 3 SERIES | 15-20 REPS |
| Leg raises | 3 SERIES | 30 SECONDS |
| Unilateral curl biceps (5kg) | 3 SERIES | 15-20 REPS |
| Wall push | 3 SERIES | 40 SECONDS |
| Dead lift (5 kg) | 3 SERIES | 10-12 REPS |
| Plank up-downs | 3 SERIES | 10-12 REPS |
| CALM DOWN | | |

| WEEK 8 (REPEAT 5 DAYS THE TRAINING) | | |
| --- | --- | --- |
| WARM UP | | |
| Jumping rope | 3 | 15-20 |
| Squat | 3 | 15-20 |
| Lunge with overhead press (5 kg) | 3 | 15-20 |
| Plank | 3 | 30 |
| High Knees | 3 | 15-20 |
| Boxing | 3 | 40 |
| Kettkebell swing | 3 | 15-20 |
| Glute bridge | 3 | 15-20 |
| Burpee | 3 | 15-20 |
| CALM DOWN | | |
