# Supplementary material for: Effects of active action observation on cognitive, emotional, motor, and somatosensory outcomes in adolescents with juvenile idiopathic arthritis: a prospective exploratory case series
Source: Front Hum Neurosci. 2026 Feb 27;20:1766070. doi: 10.3389/fnhum.2026.1766070 (PMC12982409; doi:10.3389/fnhum.2026.1766070)
Supplement: Supplementary file 1 [file Supplementary_file_1.zip › Supplementary Material/Supplementary Material 3.docx]

COOL DOWN

| Lying supine, hands on the ribs. | 5 deep breaths |
| --- | --- |
| Arms alongside the body, right fist clenched. | We hold the strength for 10 seconds, then relax for 20 seconds. |
| Arms alongside the body, left fist clenched. | We hold the strength for 10 seconds, then relax for 20 seconds. |
| Right fist clenched, we press the arm against the mat. | We hold the strength for 10 seconds, then relax for 20 seconds. |
| Left fist clenched, we press the arm against the mat. | We hold the strength for 10 seconds, then relax for 20 seconds. |
| We try to make a fist with the right foot, pressing with the toes. | We hold the strength for 10 seconds, then relax for 20 seconds. |
| We try to make a fist with the left foot, pressing with the toes. | We hold the strength for 10 seconds, then relax for 20 seconds. |
| We try to make a fist with the right foot by pressing with the toes, while at the same time pressing the leg against the floor. | We hold the strength for 10 seconds, then relax for 20 seconds. |
| We try to make a fist with the left foot by pressing with the toes, while at the same time pressing the leg against the floor. | We hold the strength for 10 seconds, then relax for 20 seconds. |
| We return with our hands on the ribs and take deep breaths. | 5 deep breaths |

These exercises will be performed in a calm environment while maintaining deep breathing.

During the relaxation period, patients must focus on the fatigue and tiredness that the muscle has accumulated after the contraction.
