# Supplementary material for: Effects of active action observation on cognitive, emotional, motor, and somatosensory outcomes in adolescents with juvenile idiopathic arthritis: a prospective exploratory case series
Source: Front Hum Neurosci. 2026 Feb 27;20:1766070. doi: 10.3389/fnhum.2026.1766070 (PMC12982409; doi:10.3389/fnhum.2026.1766070)
Supplement: Supplementary file 1 [file Supplementary_file_1.zip › Supplementary Material/Supplementary Material 4.docx]

WEEK 1

|  | Patient 1 | Patient 2 | Patient 3 | Patient 4 | Patient 5 | Patient 6 | Patient 7 | Patient 8 | Patient 9 | Patient 10 |
| --- | --- | --- | --- | --- | --- | --- | --- | --- | --- | --- |
| “Have you felt any discomfort while performing the exercises?” | NO | NO | NO | NO | NO | NO | NO | NO | NO | YES |
| “Have you had muscle soreness after doing the exercises?” | YES | YES | NO | NO | YES | NO | NO | NO | NO | NO |
| "Are you enjoying the treatment?" | YES | YES | YES | YES | YES | YES | YES | YES | NO | YES |
| "Is there any specific exercise that bothers you?" | NO | NO | NO | NO | NO | NO | NO | NO | NO | Ketelbell swing |
| "Where does it hurt?" | NO | NO | NO | NO | NO | NO | NO | NO | NO | LUMBAR |
| “Could you describe a bit what the discomfort feels like?” | NO | NO | NO | NO | NO | NO | NO | NO | NO | Like Little pricks |

WEEK 2

|  | Patient 1 | Patient 2 | Patient 3 | Patient 4 | Patient 5 | Patient 6 | Patient 7 | Patient 8 | Patient 9 | Patient 10 |
| --- | --- | --- | --- | --- | --- | --- | --- | --- | --- | --- |
| “Have you felt any discomfort while performing the exercises?” | NO | NO | NO | NO | YES | NO | NO | NO | NO | NO |
| “Have you had muscle soreness after doing the exercises?” | YES | NO | NO | YES | YES | NO | NO | NO | YES | YES |
| “Are you enjoying the treatment?” | YES | YES | YES | YES | YES | YES | YES | YES | YES | YES |
| “Is there any specific exercise that bothers you?” | NO | NO | NO | NO | The knees exercise | NO | NO | NO | NO | NO |
| “Where does it hurt?” | NO | NO | NO | NO | The knees | NO | NO | NO | NO | NO |
| “Could you describe a bit what the discomfort feels like?” | NO | NO | NO | NO | It´s a weird sensation | NO | NO | NO | NO | NO |

WEEK 3

|  | Patient 1 | Patient 2 | Patient 3 | Patient 4 | Patient 5 | Patient 6 | Patient 7 | Patient 8 | Patient 9 | Patient 10 |
| --- | --- | --- | --- | --- | --- | --- | --- | --- | --- | --- |
| “Have you felt any discomfort while performing the exercises?” | NO | YES | NO | NO | NO | NO | NO | NO | NO | NO |
| “Have you had muscle soreness after doing the exercises?” | NO | NO | NO | YES | YES | NO | NO | NO | YES | YES |
| “Are you enjoying the treatment?” | YES | YES | YES | YES | YES | YES | YES | YES | YES | YES |
| “Is there any specific exercise that bothers you?” | NO | YES, The push ups | NO | NO | NO | NO | NO | NO | NO | NO |
| “Where does it hurt?” | NO | The wirst | NO | NO | NO | NO | NO | NO | NO | NO |
| “Could you describe a bit what the discomfort feels like?” | NO | Not strong enought to mantein my weight | NO | NO | NO | NO | NO | NO | NO | NO |

WEEK 4

|  | Patient 1 | Patient 2 | Patient 3 | Patient 4 | Patient 5 | Patient 6 | Patient 7 | Patient 8 | Patient 9 | Patient 10 |
| --- | --- | --- | --- | --- | --- | --- | --- | --- | --- | --- |
| “Have you felt any discomfort while performing the exercises?” | NO | NO | NO | NO | NO | NO | NO | NO | NO | NO |
| “Have you had muscle soreness after doing the exercises?” | YES | YES | NO | YES | NO | NO | NO | NO | YES | NO |
| “Are you enjoying the treatment?” | YES | I LOVE IT | YES | YES | YES | YES, A LOT | YES | YES | A Little less | YES |
| “Is there any specific exercise that bothers you?” | NO | NO | NO | NO | NO | NO | NO | NO | NO | NO |
| “Where does it hurt?” | NO | NO | NO | NO | NO | NO | NO | NO | NO | NO |
| “Could you describe a bit what the discomfort feels like?” | NO | NO | NO | NO | NO | NO | NO | NO | NO | NO |

WEEK 5

|  | Patient 1 | Patient 2 | Patient 3 | Patient 4 | Patient 5 | Patient 6 | Patient 7 | Patient 8 | Patient 9 | Patient 10 |
| --- | --- | --- | --- | --- | --- | --- | --- | --- | --- | --- |
| “Have you felt any discomfort while performing the exercises?” | NO | NO | NO | NO | NO | NO | NO | YES | YES | NO |
| “Have you had muscle soreness after doing the exercises?” | NO | NO | NO | NO | NO | NO | NO | NO | YES | YES |
| “Are you enjoying the treatment?” | YES | YES | YES | YES | YES | YES | YES | YES | A LITTLE BIT YES A LITTLE BIT NOT | YES |
| “Is there any specific exercise that bothers you?” | NO | NO | NO | NO | NO | NO | NO | The exercise that stretch the leg | SQUAT, AND JUMPS WITH BUT KICKS | NO |
| “Where does it hurt?” | NO | NO | NO | NO | NO | NO | NO | The knee | GROIN AND GASTROCNEMIOUS | NO |
| “Could you describe a bit what the discomfort feels like?” | NO | NO | NO | NO | NO | NO | NO | Like a prick | Like a prick | NO |

WEEK 6

|  | Patient 1 | Patient 2 | Patient 3 | Patient 4 | Patient 5 | Patient 6 | Patient 7 | Patient 8 | Patient 9 | Patient 10 |
| --- | --- | --- | --- | --- | --- | --- | --- | --- | --- | --- |
| “Have you felt any discomfort while performing the exercises?” | NO | NO | NO | NO | NO | NO | NO | NO | NO | NO |
| “Have you had muscle soreness after doing the exercises?” | NO | NO | NO | NO | NO | YES, in arms | NO | NO | YES, A LOT | YES |
| “Are you enjoying the treatment?” | YES, BUT NOT TO MUCH | YES | YES | YES | YES | YES | YES | YES | NOT AT ALL | YES |
| “Is there any specific exercise that bothers you?” | NO | NO | NO | NO | NO | NO | NO | NO | NO | NO |
| “Where does it hurt?” | NO | NO | NO | NO | NO | NO | NO | NO | NO | NO |
| “Could you describe a bit what the discomfort feels like?” | NO | NO | NO | NO | NO | NO | NO | NO | NO | NO |

WEEK 7

|  | Patient 1 | Patient 2 | Patient 3 | Patient 4 | Patient 5 | Patient 6 | Patient 7 | Patient 8 | Patient 9 | Patient 10 |
| --- | --- | --- | --- | --- | --- | --- | --- | --- | --- | --- |
| “Have you felt any discomfort while performing the exercises?” | NO | YES, In groins | NO | NO | NO | NO | NO | NO | NO | NO |
| “Have you had muscle soreness after doing the exercises?” | YES | YES, A LOT | NO | NO | NO | NO | NO | NO | YES | YES |
| “Are you enjoying the treatment?” | NOT AT ALL | YES | YES | YES | YES | YES | YES | YES | NOT AT ALL | YES |
| “Is there any specific exercise that bothers you?” | NO | In leg raises | NO | NO | NO | NO | NO | NO | NO | NO |
| “Where does it hurt?” | NO | In abds | NO | NO | NO | NO | NO | NO | NO | NO |
| “Could you describe a bit what the discomfort feels like?” | NO | It seems like i can´t breath | NO | NO | NO | NO | NO | NO | NO | NO |

WEEK 8

|  | Patient 1 | Patient 2 | Patient 3 | Patient 4 | Patient 5 | Patient 6 | Patient 7 | Patient 8 | Patient 9 | Patient 10 |
| --- | --- | --- | --- | --- | --- | --- | --- | --- | --- | --- |
| “Have you felt any discomfort while performing the exercises?” | NO | NO | NO | NO | NO | NO | NO | NO | NO | NO |
| “Have you had muscle soreness after doing the exercises?” | YES | YES | NO | NO | NO | NO | NO | YES | YES, A LOT | YES |
| “Are you enjoying the treatment?” | YES | YES | YES | YES | YES | YES, A LOT | NOT AT ALL | YES | I DON´T MIND | YES |
| “Is there any specific exercise that bothers you?” | NO | NO | NO | NO | NO | NO | NO | NO | NO | NO |
| “Where does it hurt?” | NO | NO | NO | NO | NO | NO | NO | NO | NO | NO |
| “Could you describe a bit what the discomfort feels like?” | NO | NO | NO | NO | NO | NO | NO | NO | NO | NO |
